# Finding the Return Path: Landmark Position Effects and the Influence of Perspective

**DOI:** 10.3389/fpsyg.2016.01956

**Published:** 2016-12-23

**Authors:** Harun Karimpur, Florian Röser, Kai Hamburger

**Affiliations:** ^1^Experimental Psychology and Cognitive Science, Justus Liebig University GiessenGiessen, Germany; ^2^Department of Social Sciences, University of Applied SciencesDarmstadt, Germany

**Keywords:** wayfinding, navigation, spatial cognition, return path, landmarks, egocentric, allocentric, structural salience

## Abstract

Much research has been done on how people find their way from one place to another. Compared to that, there is less research available on how people find back from the destination to their origin. We first present theoretical approaches to perceptual and cognitive processes involved in finding a return path, including concepts, such as visibility, structural salience, and allocentric versus egocentric perspective, followed by a series of three experiments. In these experiments, we presented subjects intersections that contained landmark information on different positions. In order to investigate the processes involved, we used different measures, such as route-continuation (in learning direction and in opposite direction) and free-recall of route information. In summary, the results demonstrate the importance of landmark positions at intersections (structural salience in combination with perspective) and that finding the return path is more difficult than reproducing the same route from the learning condition. All findings will be discussed with respect to the current research literature on landmark-based wayfinding.

## Introduction

In this study, we are going to present findings from the field of human wayfinding. Finding our way is a complex endeavor, which consists of many building blocks (e.g., features of reference points, working memory, etc.). In order to do justice to the facets of this area, this complexity will be reflected by the structure of this paper. Here, we want to focus on three problems, which we will introduce in the first part; these are perspective, return path, and landmark position. After, we will postulate our theoretical assumptions. Then, in a series of three experiments, we will approach these problems from different angles. Finally, these findings are being discussed in the light of the current literature.

### The Right Perspective

Imagine that you are on a vacation in an unknown foreign city. After your arrival at the hotel you want to explore the surroundings and maybe visit a place of interest or a touristic feature (e.g., a famous building, such as the Eiffel Tower in Paris). How do you proceed?

You may want to use a verbal description that you received at the reception desk of your hotel. Maybe you want to make use of a city map a tourist guide gave you. Or, if you do not have these means at hand, you may want to ask a pedestrian on the street for giving you directions to your goal location. These examples show that different perspectives are involved. In route learning, we differentiate between an egocentric perspective (e.g., verbal description from the first person view) and allocentric perspective (e.g., a map). One important question is whether the verbal description is sufficient on its own for reaching the goal location. Or, would it be better to supplement the verbal description with a map, or maybe make only use of the map instead? This is what we will call: Problem 1 – the problem of perspective. This question does not only address the issue of getting lost (Dudchenko, 2010) but also the idea of cognitive economy, namely, reaching the goal with the least cognitive or physical effort.

### Finding Our Way Back

So, let us assume that we successfully reached the goal in our initial example. We are now faced with a new, more difficult problem. We need to return to our origin and preferably on the easiest, fastest, and/or most economical way (= Problem 2; return path).

The nature of homing behavior in animals has been studied well over the course of the last decades. From some insects it is known that they switch to a landmark-based (compared to a vector-based) wayfinding strategy when landmarks are available. Both strategies can be used independently from one another but landmarks can help to gauge vectors in the long run (Collett and Collett, 2000). In other experiments, desert ants learned a path to their nest that consisted of landmarks along the route. When the home vector did not match with the array of landmarks, about half of the ants navigated home by relying on the home vector (Wehner, 2003).

In human navigation, finding a return path is an everyday problem, which, however, has been treated with less attention in the past. Often scientists refer to strategies like *retrace strategy* (e.g., Golledge, 1995), *path integration* (e.g., Wiener et al., 2011; Mallot, 2012) or *look-back strategy*:

“[…] the look-back strategy involves intentionally stopping, turning around, and memorizing the view behind you while traveling along a route. The […] traveler sometimes does not recognize the view in the reverse direction and makes a wrong choice.” (Montello, 2005, p. 270).

Cornell et al. (1992) investigated finding the return path and compared such strategies with different age groups. Their findings suggest that especially the look-back strategy can be helpful. In a study focusing on aging, Wiener et al. (2012) showed that older subjects have more problems with the return path compared to younger subjects. The authors suggest that allocentric processing (which is thought to be impaired in the elderly) plays an important role in route retracing.

Nevertheless, it seems clear that we are able to manage this task, but we do not yet know the underlying cognitive and neural processes enabling us to find the return path. For an artificial cognitive system this task may be easier, since inverting the learned direction sequences and distances can be handled without any distraction or error. It is important to note that returning on the same route is, cognitively speaking, much different from path integration or *spatial updating*, e.g., when a road is blocked and we need to find a detour (for an extensive overview including comparative studies see Dudchenko, 2010).

### Landmarks and Their Position

In general, wayfinders use so-called landmarks, objects or buildings that stand out from the environment (e.g., Lynch, 1960; Presson and Montello, 1988; Raubal and Winter, 2002; Caduff and Timpf, 2008; an extensive overview about different modern approaches on landmarks can be found in Richter and Winter, 2014). The recognition of landmarks is important because, in the long run, they can be associated with a certain direction and movement toward the direction (Trullier et al., 1997; Denis et al., 2014). The extent to which the landmark stands out from the environment is described with the term landmark salience. A look into the general literature on landmark-based wayfinding reveals several approaches, i.e., the importance of an object at certain locations in order to aid successful navigation (e.g., Lynch, 1960; Presson and Montello, 1988; Sorrows and Hirtle, 1999). The majority of landmark models define landmark salience as inherent features of an object or intersection. In contrast, Caduff and Timpf (2008) focus on the observer with her cognitive abilities and limitations in order to provide a more observer-based landmark salience approach.

Other studies emphasize the distinction between landmarks as associative cues and beacons (e.g., Waller and Lippa, 2007). The former function describes the case where a landmark (e.g., a statue at an intersection) is associated with a certain direction (e.g., turn left). The latter function describes a landmark that can be seen as a goal (or is close to a goal). In our example, by looking at the statue, we might begin to move toward the statue and do the same with the subsequent landmarks. It seems as if these beacon strategies are more economic in the sense that less information needs to be encoded for finding our way. It is noteworthy that there seems to be a connection between the theory of beacon strategies and structural aspects (landmark position). However, this was not the focus of the current study.

**FIGURE 1:**
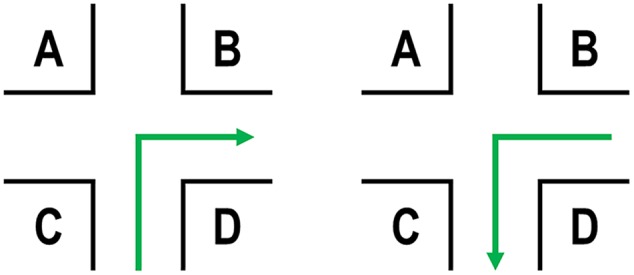
**Initial path (left) and the same intersection for the return path (right) from an allocentric perspective.** Landmark position connotations are defined according to the initial path.

One important aspect for the return path is the arrangement of the environment (= Problem 3; landmark position), i.e., structural landmark salience (Sorrows and Hirtle, 1999; Klippel and Winter, 2005). We assume visual salience – that is how much an object stands out from its immediate surrounding (e.g., Caduff and Timpf, 2008) – and semantic salience of landmarks – that is for example its name, meaning, or function (Raubal and Winter, 2002; Nothegger et al., 2004; Hamburger and Knauff, 2011) – to be less important than the structural salience (Hamburger and Röser, 2011, 2014; Röser et al., 2012a). Therefore, we here try to control for these aspects and rather focus on the structural aspects as we have done in previous experiments on structural salience in which we used a route-continuation paradigm (Röser et al., 2012a,b). In this respect, the following ideas are based on a simple intersection with four potential landmark positions (**Figure 1**), which we will hold constant throughout the manuscript. Please note that the landmark position connotations are in accordance with the initial path; this can be neglected for positions *A* and *D* (invariant), but is important for positions *B* and *C*. Further details on this theoretical assumption will be provided in the following section.

## Theoretical Assumptions on the Return Path

In the first section, we described three problems: (1) perspective, (2) return path, and (3) landmark position. In the following, we try to connect these problems by presenting current ideas on how landmarks, places, and directions might be cognitively processed for the return path. Within this scope we would like to address the aforementioned problems in greater depth on a theoretical level.

Why does (1) perspective matter when we talk about (2) the return path or (3) landmark position effects (structural salience)? The reason for this is that the position preference is dependent on both the position of the observer (Röser, 2015) and the observer’s encoding perspective where many researchers differentiate between an egocentric (self-to-object) and allocentric (object-to-object) perspective (Bryant, 1997; Klatzky, 1998; Nadel and Hardt, 2004; Coluccia et al., 2007).

We here define allocentric as a birds-eye or map perspective, so that the information is seen from above (survey information) and has the same visibility for all parts of an intersection. Egocentric is here defined as the body-centered view of an agent standing in the environment, including different visibilities at an intersection (e.g., Winter, 2003; Röser, 2015).

As mentioned, it seems to be important to understand the influence of different encoding perspectives in order to understand landmark position preferences. Both learning from maps (i.e., allocentric presentation) and learning from navigation (i.e., egocentric presentation) received much attention in the literature. For example, Thorndyke and Hayes-Roth (1982) examined which of both learning modalities is better suited for acquiring route knowledge and reported mixed findings (e.g., navigation-learning subjects were better in estimating route distances but not in judging the relative location of objects). These findings are crucial and could also explain so-called switch-costs. For instance, when encoded in egocentric perspective, recognition is more difficult in allocentric perspective (Shelton and McNamara, 2004). It further demonstrates a common problem: supra-modal spatial representations are not necessarily indicative of equal suitability for information retrieval. The mere existence of a cognitive map would not guarantee a null effect between initial and return path. For example, a recent study (carried out in a parking lot of a shopping mall) showed that the return path was on average 10% longer than the initial path (Mora et al., 2014).

Because of the above findings, we implemented the role of perspective and the return path into our experimental designs. As previously mentioned, such factors are important in order to examine the role of position effects in human wayfinding, or in a more general sense: the structural landmark salience. In the following, we postulate our theoretical assumptions on this topic with respect to allocentric perspective, egocentric perspective and direction specificity.

### Allocentric Perspective

In the allocentric perspective, on the forward run, the optimal position (Klippel and Winter, 2005; Röser et al., 2012a,b; Röser, 2015) is *D, before the intersection and in direction of turn*. This has been suggested theoretically/mathematically (Klippel and Winter, 2005) and has been evaluated empirically (Röser et al., 2012a,b). The importance of landmarks being located in direction of a turn can also be explained by the use of a beacon strategy (Waller and Lippa, 2007). It allows us to move *towards* a landmark and thus spares us encoding associated directions. The importance of location *D* for route learning could also be shown in recent eye-tracking studies (de Condappa and Wiener, 2016).

**FIGURE 2:**
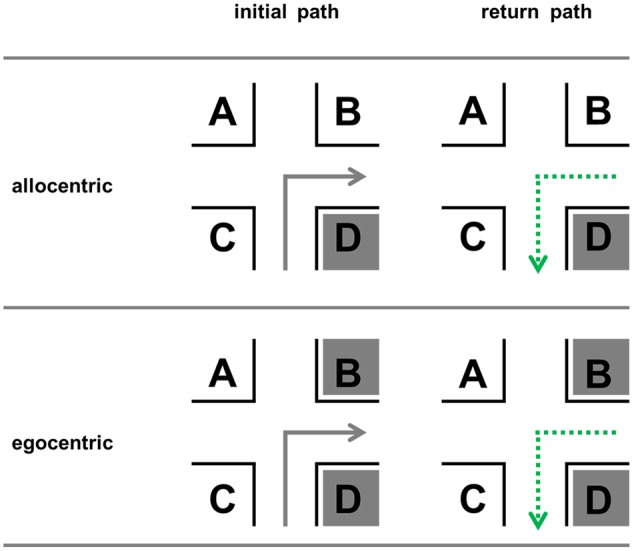
**Possible optimal (dark gray) landmark positions for the forward run and the return path in the allocentric and egocentric perspective**.

For the return path the optimal positions are not yet known. We assume that it remains to be *D* since this location is still *before the intersection* and *in direction of turn* (**Figure 2**). This position is invariant, i.e., independent of traveling direction; no right/left encoding is necessary.

### Egocentric Perspective

**FIGURE 3:**
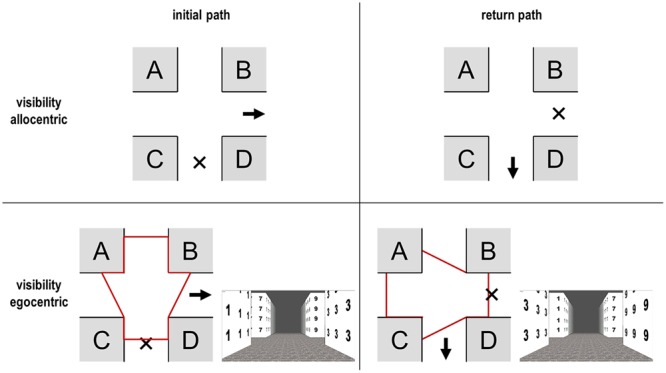
**Visibility from two different positions: initial path (left) and return path (right)**. “X” = position of individual; “→” = walking direction. In the allocentric perspective each position is equally visible for both directions, not so for the egocentric perspective. The red-framed area indicates the visible parts of the path, while the small images on the bottom visualize the sight in the egocentric perspective.

An important issue in the egocentric perspective is the so-called “visibility” (Winter, 2003; Röser et al., 2012b). This means that a change of the view direction or the position of the observer influences the visible parts of the scene (**Figure 3**).

Visual attention (bottom-up and top-down) is generally paid to the direction of turn (e.g., Itti and Koch, 2000; Jin et al., 2004). It seems that in an egocentric perspective it is important that a landmark is at least located *in direction of turn* and that exact positions *before* and *behind* become less important (Röser et al., 2012a,b). For the return path, it is important to take the visibility *and* structural aspects into account. According to the above findings and the previous logic, the optimal positions in the egocentric perspective could also be *C* and *D*, since they are in direction of the turn on the return path. However, information can only be retrieved after successful encoding. In other words: the structural arrangement during encoding is decisive. We could conclude that position *C* was suboptimal on the forward run and therefore it may now be doubted that it really becomes optimal on the return path.

Now it is interesting to see that positions *D* and *A* are invariant for the initial and the return path. For example, *D* will remain the position *before the intersection in direction of turn* in both the initial and the return path. Positions *B* and *C* are variant locations because they have to be mentally and verbally transformed for the return path. For example, *B* is the position *behind the intersection in direction of turn* for an initial path. And it will then turn to the position *before the intersection opposite to the direction of turn* on the way back. But, this is only the case if the spatial information is *unspecific*: “turn into direction of D” or “turn in the opposite direction of A”. If the information is direction *specific*, i.e., right and left, then right has to be cognitively transformed into left on the return path and vice versa (see the section Direction Specificity).

According to the concept of visibility (Winter, 2003; Röser et al., 2012a,b), it is furthermore important in the egocentric perspective that both facades at one location at the intersection are visually identical/similar (e.g., same color and texture) if only one is visible. Of course, this only holds on a theoretical level because it is rarely the case and agents could also wait for reaching the middle of an intersection. If for instance one facade is brown and the other white, then there is nothing that can be recognized on the return path (in theory), since one of them was not seen before (**Figure 3**, positions *C* and *D* with numbers). For instance, if both facades are similar, then this information can be used for the return path, but if they differ significantly, then position *D* becomes useless on the return path, since it cannot be recognized anymore. It may then only be recognized if the observer turns the head on the initial path at the intersection at a point in time when the route decision has already been made (look-back strategy). Objects must therefore be recognizable; see **Table 1** for theoretical predictions; please note the lower right value, which has the most dramatic effects depending on visibility and equal appearance.

**Table 1:** Visibilities for the different landmark positions (A–D) in **Figures 2** and **3** for the initial and the return path given that both facades of a corner are same looking, and for the return path in case of different facades; 0 indicates that no facade is visible, 0.5 indicates that one facade in visible, and 1 means that both possible facades of a building at an intersection are visible and therefore contain the maximum of information available.

Path/position	Initial path	Return path (same facade)	Return path (different facades)
A	1	1	1
B	1	0.5	0.5
C	0.5	1	0.5
D	0.5	**0.5**	**0.0**

*Here, both facades of a single building have the same characteristics/appearance. Thus, position D has a visibility of 0.5 on the return path, since the visible facade is similar to the one seen on the initial path. When two facades of each building are different in their appearance, the visibility of D becomes 0, since here the new facade on the return path does not contain any information about this position compared to the initial path.*

### Direction Specificity

**FIGURE 4:**
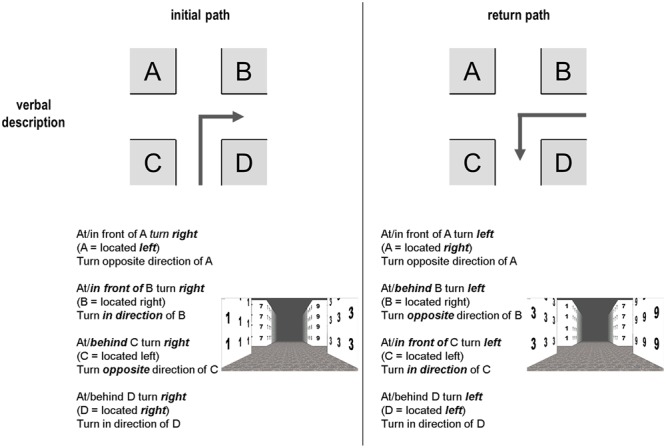
**Examples for verbal directions in the forward run and the return path.** Note that the descriptions for positions *D* and *A* do not change, while large changes occur for positions *C* and *B*.

Not only the visibility represents an important issue but also language and how it is used when giving directions, learning new pathways, and mentally transforming them for the return path. As mentioned above, there are at least two ways of spatial directions: *direction specific* and *direction unspecific* verbal information (**Figure 4**).

Direction specific here means that a precise direction with a single spatial word is provided, e.g., *right/left or east/west*. At first glance this information is easy to understand and simple to use. But, it becomes complicated if the return path has to be constructed, since then a *left turn* needs to become a *right turn* and vice versa. Thus, an additional mental transformation is required. This is not necessary if a direction unspecific representation is used, without spatial directions but rather based on landmark locations (tested in a wayfinding experiment; Bucher et al., 2014). In other words, the verbal direction *turn in the direction of the gas station* does not need to be mentally or verbally transformed if it is located on position *D*; the same is true for position *A* with the instruction *turn in opposite direction of A*. On the return path, both locations and unspecific directions would remain the same: in the mental representation the gas station would still either be *in direction of turn* (*D*) or *opposite to the direction of turn* (*A*). This would require one mental processing step less, since no transformation would be required (left → right) resulting in less cognitive load. But, is this how wayfinders encode spatial information and directions? It would at least make much sense from an economical perspective. For the return path, we therefore assume that *direction unspecific* information would be less effortful and therefore preferable over a *direction specific* strategy that, in theory, results in higher cognitive load. However, we do not believe that *A* represents an optimal landmark position when no description is given. The importance of a landmark being in direction of the turn during encoding remains the most important factor (Röser, 2015). We therefore assume that *B* and *D* will be optimal positions for the egocentric perspective also for the return path when no verbal description is given.

### Aim of the Study

As demonstrated, we are faced with different problems. In order to ensure that this study reflects the complexity of each of the above problems, it is important to examine this topic from different angles. This requires us to switch the experimental paradigm from experiment to experiment. Nevertheless, all experiments are designed to contribute to the common goals of this study. The designs are based on simple blocks world maze similar to those used by many other researchers in this area (e.g., Janzen and van Turennout, 2004; Newman et al., 2007; Stankiewicz and Kalia, 2007; Wiener et al., 2012). In each experiment, we will consider the question of the return path (Problem 2) and optimal landmark positions (Problem 3) while using different methods. With each experiment, we will more and more focus on the problem of perspective (Problem 1), that is how perspective affects our abilities to find our way in an unfamiliar environment. We decided to proceed as follows:

Our first two experiments are wayfinding experiments in which we placed landmarks on one of the four positions (*A*–*D*). In Experiment 1, we systematically investigate how wayfinders encode given (unfamiliar) routes and how transforming them into a return path affects their wayfinding performance. In Experiment 2, we begin to address how different learning strategies or perspectives (verbal description versus map learning) influence our wayfinding performance. Finally, in Experiment 3, we place landmarks on all of the four positions and examine position preferences. We will show that these preferences not only differ between initial and return path but also between egocentric and allocentric perspective when using free recall and not just cued recall.

## Experiment 1 – Initial Path versus Return Path

In the first experiment, wayfinding performances when finding the initial and the return path are compared with each other.

### Method

#### Subjects

A total of 20 Psychology students from the University of Giessen participated (16 females and 4 males). They had a mean age of 23.5 years (*SD* = 4.08). All subjects were naive with respect to this study, provided informed written consent, and received course credits for participation. They had normal or corrected-to-normal visual acuity and were free of any pre-existing psychiatric or neurologic illness (e.g., epilepsy).

#### Materials

The equipment included a custom 19” monitor (Dell), a Personal Computer (HP Compaq 6000 Pro), and a Response Pad (RB-530 Cedrus Corporation©). For presentation and data recording SuperLab 4.0 Stimulus Presentation Software (Cedrus Corporation©) was employed.

**FIGURE 5:**
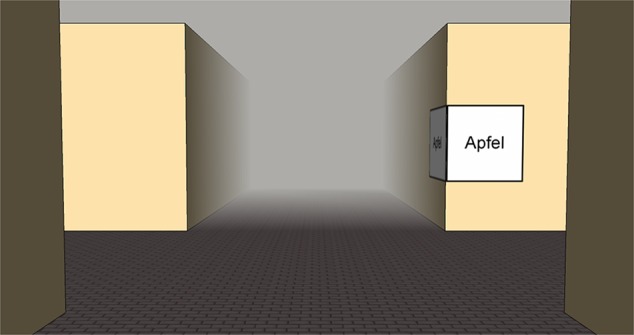
**Screenshot of an intersection in the virtual maze (decision point).** The landmark (word; “Apfel” = apple) is presented on both facades at one corner (position) in order to provide recognizability on the return path (compare with **Table 1**).

The virtual environment (maze) was set up with Google© SketchUp 8 (compare to SQUARELAND; Hamburger and Knauff, 2011). Here, 24 routes, each with eight intersections in an egocentric perspective, were created. The directions left or right were used and every intersection contained only one landmark – a word on a white sign (**Figure 5**).

Every intersection (24 × 8 = 192) contained one distinct landmark. Hence, a landmark which was shown once to a subject did not appear again later in another route. We controlled for all landmarks being comparably imaginable by using familiar, everyday words (e.g., tiger, salt, guitar, church, and banana). A landmark was placed on both sides of the corresponding facades of a corner, so it would be visible and completely readable from both directions of travel – forward and backwards. They were presented at a simulated eye height of 170 cm with the same distance to the center of the intersections.

To control for direction or landmark position effects, the number of right/left turns and the position of landmarks (before or after the intersection, in or against moving direction) were balanced for single routes.

#### Procedure

Subjects learned a route of eight intersections via successively presented images of each of the intersections (**Figure 5**). Every intersection was shown for duration of eight seconds (learning phase). With each intersection verbal information was presented to indicate the correct direction (e.g., “turn right”). Subsequently, subjects were instructed to find the same path again (wayfinding phase) either in the normal (forward from origin to destination) or the reverse travel direction (backwards from destination to origin). Every intersection was again presented via pictures from the corresponding point of view – please note that the perspective of the previously learned intersections changed when the return path had to be found – and served as a decision point (right or left) for which direction decisions had to be made. On each decision point, subjects had to indicate the correct route by pressing a corresponding button. The correct route (screenshot sequence in walking orientation) was continued independent of which direction was chosen. Thus, no feedback was provided in this experiment.

After one route was navigated (eight direction decisions), the learning phase of the next route started. The total of 24 routes had to be learned by each subject. Overall, half of the routes had to be found in the forward run direction, while for the other half the return path was required. Therefore, two experimental versions were used where navigation direction in the wayfinding phase was interchanged (e.g., Route 1 had to be found again in forward direction in version 1, but in the backwards direction in version 2). The order of the routes was randomized across subjects. Correct decisions and response times served as dependent variables.

### Results

**FIGURE 6:**
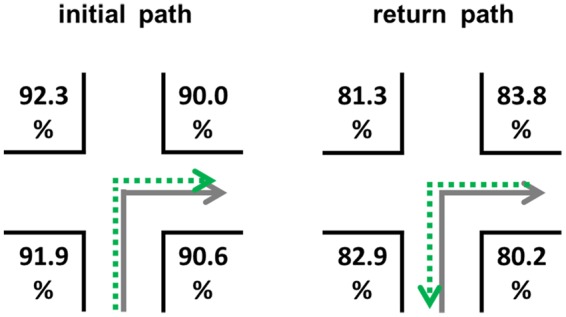
**Correct route decisions according to the four different landmark locations (*A–D*) on the initial and return path**.

The mean correct route decisions were about 86.62% (*SEM* = 2.30) in this experiment (original path: 91.20%, *SEM* = 1.92; return path: 82.03%, *SEM* = 2.99; chance level 50%). The detailed results for the positions on the return path are visualized in **Figure 6**. Mean response times were about 1910 ms (*SEM* = 192; original path: 1523 ms, *SEM* = 150; return path: 2297 ms, *SEM* = 255).

An analysis of variance with repeated measures for the wayfinding phase was performed. Within-subject factors were navigation direction (two levels: forward/backward) and landmark position (four levels: positions *A*–*D*). Both, for correct decisions and response times a significant main effect for navigation direction [correct decisions: *F*(1,19) = 19.865, *p* < 0.001; response times: *F*(1,19) = 21.571, *p* < 0.001], but not for landmark position [correct decisions: *F*(3,57) = 1.020; response times: *F*(3,57) < 1] could be found. Subjects were better and faster in navigating the original route direction (forward) compared to the reverse direction (backwards), but the position of a landmark did not lead to any performance differences.

### Discussion

An overall effect for the wayfinding direction could be found. People were faster and better when travelling the route in the originally learned direction (forward) compared to navigating the return path. This is consistent with findings using similar or even real life paradigms (e.g., Gillner and Mallot, 1998; Wiener et al., 2012; Mora et al., 2014). No landmark position effect was found.

As mentioned above, this experiment was supposed to represent an overall randomized and balanced experiment. It is therefore possible that this theoretically derived design does not represent landmark-based wayfinding, but induces other learning behaviors. The majority of subjects (65%) reported that only the direction of turn (left/right) was used as learning cue and that landmarks were rather ignored, because they did not (subjectively) aid the learning process. Also the amount of routes to memorize was criticized by some subjects. For these subjects the wayfinding task became a mere memory task for directions (serial learning; e.g., Buchner and Jansen-Osmann, 2008). This is of interest for understanding the principles of route learning and wayfinding on the one hand (at what point do people really need landmarks?), but could suppress effects regarding the structural salience of landmarks. Under these circumstances, it becomes clear why we obtained differences in regards to the return path but no general effect of landmark position.

## Experiment 2 – Egocentric Versus Allocentric Return Path

In Experiment 2, we will examine landmark position effects but also the influence of perspectives. In the section “Theoretical Assumptions on the Return Path”, we explained how the fact that a landmark position is variant (*B* and *C*) or invariant (*A* and *D*) could influence wayfinding performance. On the other hand, we also mentioned counterarguments (e.g., landmark position during encoding, i.e., initial path is more important). We therefore wanted to understand whether landmark position constancy plays a role or not. Another question addressed in this experiment was how different description strategies (verbal description from egocentric perspective – map from allocentric perspective) influence our wayfinding performance. In order to test this, we changed our experimental paradigm to a more realistic setup. Subjects were now confronted with video sequences from an egocentric perspective with approximated true physical sizes on a projection screen. We created one route but with the option of going straight, two learning conditions and increased the number of intersections (further details are given in the Method section).

It is noteworthy that introducing the option of going straight brings another potential strategy which Meilinger et al. (2014) call the *When in doubt follow your nose* strategy. It describes a strategy with which wayfinders walk straight by default and memorize turns. This in turn might lead to a reduction of number of intersections subjects need to learn. However, we decided to introduce it for three reasons. First, it is not unusual to use X-crossings in the field of spatial cognition (e.g., Janzen and van Turennout, 2004; Janzen, 2006; Janzen et al., 2008; Röser et al., 2011). Second, modern urban environments also consist of more than just two options such as left and right. Thinking of classical X-crossings, a third option should not lessen the strength of our results. Third, the patterns presented by the authors were not found for wayfinding tasks but only for planning routes.

### Method

#### Subjects

A total of 20 Psychology students from the University of Giessen participated (13 females and 7 males). They had a mean age of 26.1 years (*SD* = 9.03). All subjects were naive with respect to this study, provided informed written consent, and received course credits for participation. They had normal or corrected-to-normal visual acuity and were free of any pre-existing psychiatric or neurologic illness (e.g., epilepsy).

#### Materials

The same setup as in Experiment 1 was used, except for a customary projection screen (171 cm × 238 cm) with a projector (Panasonic PT-F100NT). In this experiment, two different routes (route A and route B) through the maze were created. Each route contained 20 intersections with one landmark on either an invariant (*A, D*) or variant (*B, C*) position (eight left, eight right, and four straight ahead; the latter ones served as controls). Therefore, a total of 40 different words served as landmark objects (**Figure 5**). The words were derived from a catalog of pictograms in order to ensure that the written words we used were easy to imagine. In the maze, the landmarks were again placed on both facades of a corner (position), so that they were visible from both directions of travel.

**FIGURE 7:**
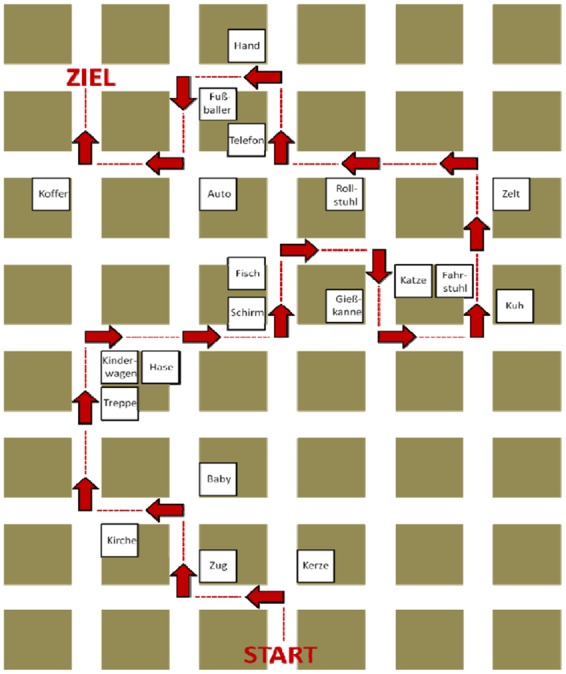
**Map condition in Experiment 2**. Subjects had to learn the route from start (“START”) to the goal location (“ZIEL”). The route was highlighted via dotted red lines and arrows, and the landmark words were located at the different positions (*A*–*D*) at the intersections.

Subjects received a map or a verbal description to learn the route (between-subjects factor). The verbal description was written from an egocentric perspective whereas the map was presented in form of an allocentric perspective. In the verbal condition, we employed sentences like: “Turn left at the apple” or “Turn right at the chair”. Wording and structure of the sentences were kept similar. The allocentric maps showed the relevant section instead of the whole SQUARELAND maze. The route was highlighted by red arrows and a dotted red line. To assure that subjects learned the route in the right direction the words “start” and “goal” marked the starting point and the end of the route (**Figure 7**).

Videos of the return path of the two routes were generated from an egocentric perspective, with an eye height of 1.70 m and a constant walking speed of about 2 m/s. For presentation and data recording SuperLab 4.0 Stimulus Presentation Software (Cedrus Corporation©) was employed.

#### Procedure

Subjects were randomly assigned to one of the two groups: One of them learned a path with 20 intersections via a map (allocentric learning condition), the other one through verbal description (egocentric learning condition). They had three minutes to encode the routes. The subjects were not aware that they would have to recall the return path instead of the initially studied route. After a five minute break, the learned path was shown as video in reverse order through the virtual maze. It was the first time the subjects came to know their real task: finding the return path (wayfinding phase). The video stopped at every intersection (decision point, always the same distance to the middle of the intersection and to the walls at the side) for subjects to indicate the path directions right, left, or straight. After a wrong decision, the video continued in the right direction so that each subject was able to complete the route (implicit feedback). But, no explicit feedback about the performance was given. Learning condition (map; verbal description) and landmark position (invariant *A* and *D*/variant *B* and *C*) served as independent variables while correct route decisions and response times served as dependent variables. The categorization of a position into optimal and suboptimal was based on the theoretical assumptions made in the Section “Theoretical Assumptions on the Return Path.”

### Results

With landmarks being located in invariant positions *A* and *D*, correct decisions on the return path were made in about 67.5% (chance level 33.3%) if the initial path was learned via a verbal description. When the path was encoded via a map, about 65.0% correct route decisions were made. With landmark objects being in variant positions *B* and *C*, on the return path, 58.8% correct decisions were made for the verbal description condition and 57.5% for the map condition.

For the invariant positions the response times were shorter (3900 ms) in the verbal description condition (egocentric), compared to the allocentric map condition (4960 ms). Responses for intersections with landmarks on variant positions revealed a shorter response time for the verbal description condition (4175 ms), in comparison to the map condition (4825 ms).

An analysis of variance with the within-subject factor landmark position (invariant/variant) and the between-subject factor learning condition (map/verbal description) was performed. It revealed a significant main effect of position on correct decisions [*F*(1,18) = 4.99, *p* = 0.038] but not on response times [*F*(1,18) < 1]. The different learning conditions did neither differ significantly with respect to correct decisions nor with respect to response times [all *F*(1,18) < 1]. The three possible route directions on the intersections (left, right, and straight on) did not lead to significant differences in regards to correct decisions [*F*(2,38) < 1]. No interactions were obtained.

### Discussion

The current experiment investigated the influence of landmark position and learning modality on finding the return path. The landmark position led to significant differences in performance (correct decisions), while this was not the case for the decision times. More correct decisions were made if landmarks were located on invariant positions. Since no decision time differences could be obtained, this effect cannot be due to longer viewing times for the landmarks. Because in one condition verbal descriptions were used, we would have expected an interaction between landmark position and learning modality. In this experiment, we did not find such an effect.

In contrast to our other experiments in this paper, one methodological limitation was that our hypothesis driven approach led to a strict distinction between variant and invariant landmark positions. This in turn did not allow for a further distinction between the four positions. Nevertheless, the results emphasize the importance of structural landmark salience. We conclude that the extent to which a landmark can serve as a point of reference for finding the return path highly depends on its position, as has previously been assumed for the “initial path” (forward run; Klippel and Winter, 2005; Röser et al., 2012a,b; Röser, 2015).

One potential limitation could be that subjects were surprised and that in turn could have influenced there performance. However, we conducted preliminary experiments in order to strike out such an explanation. In these experiments, participants were assigned to one of four groups (between-subjects design). The groups were as follows: FF, FB, BF, BB. The first part of the group label refers to the instruction-direction (forward F or backward B). The second part of the group label refers to the test-direction (forward F or backward B). Our results showed that there was no difference between FB and BB and no difference between FF and BF meaning that only the test direction (but not the instruction itself) had an effect on wayfinding performance.

The different learning conditions map (allocentric) and verbal description (egocentric) did not lead to a significant difference in the wayfinding phase, neither for correct decisions nor for the response times. This absence of significant differences may be explained by the “dual coding theory of human wayfinding knowledge” (Meilinger et al., 2008). It assumes that environmental information is (sometimes) encoded in a spatial format alone but sometimes additionally in a propositional format. The similar performances after studying a map or a verbal description may be attributed to verbal representations existing for both encoding strategies (Meilinger and Knauff, 2008). These results are also in line with findings of a wayfinding experiment by Hamburger et al. (2012). In their experiment, they found no difference between map learners and verbal description learners for a short route. Only in a condition with a long route a superiority of maps could be found. In these experiments, subjects had to recall an initial path, so route length would be an interesting topic in combination with finding a return path. The route length discussed here might also be a reason why Experiment 1 did not show the expected results, due to a very short route and possible ceiling effects for the landmark positions.

## Experiment 3 – Landmark Location Preferences And Perspective

The aim of Experiment 3 was to show that landmark position preferences not only differ between initial and return path but also between egocentric and allocentric perspective. We decided to place landmarks on all four positions and measured subjects’ preferences by using free recall (“Which position is associated with the most correct recalls?”). Additionally, we assessed whether landmarks and directions were correctly associated. Based on our findings in Experiments 1 and 2 as well as our theoretical assumptions in the Section “Theoretical Assumptions on the Return Path,” we investigated the following three hypotheses:

• Describing the initial path will result in higher landmark and direction accuracy than describing the return path (hypothesis 1).
• The position preferences do not depend on the wayfinder’s task (describing initial versus return path (hypothesis 2).
• The described landmark positions differ between allocentric and egocentric perspective. In the allocentric encoding condition, *D* will be preferred (hypothesis 3a). In the egocentric encoding condition, positions in direction of the turn, *B* and *D*, will be preferred (hypothesis 3b).

For readability and coherence reasons, the following experiment will be divided into two sections. First we will focus on our work in regards to allocentric perspective (hereafter referred to as Experiment 3a); then, we will present our findings in regards to egocentric perspective (Experiment 3b), followed by a brief summary of these findings.

### Experiment 3a – Landmark Location Preferences from an *Allocentric* Perspective

#### Methods

##### Subjects

A total of 127 individuals (79 females, 44 males, and four did not provide gender information) participated in this online-experiment. The mean age was 23.96 years (range = 18–46). They were recruited via a circular e-mail at the Justus Liebig University Giessen. Sixty-seven percent (85 subjects) indicated to have a high-school diploma or similar. For the analysis a total of 62 could be included, since the others dropped out during the experiment and did not complete it. The remaining sample consisted of 44 females and 18 males with a mean age of 23.61 years (range = 18–32). The percentage of high-school diploma or similar increased to 74%. All subjects provided informed consent and participation was voluntary without any compensation.

##### Materials

**FIGURE 8:**
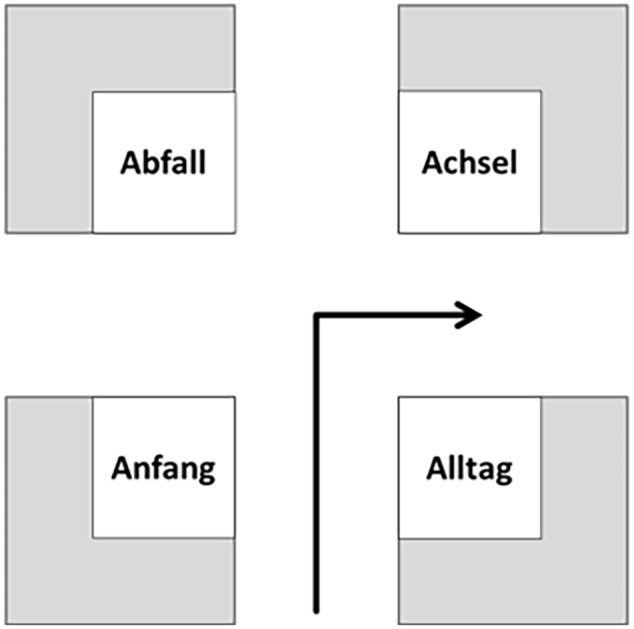
**Exemplary intersection in the allocentric perspective; four words are shown – all of them starting with the letter A in German language (Abfall = trash; Achsel = armpit; Anfang = beginning; Alltag = everyday life)**.

The experiment was run online via LimeSurvey2.05+ (Schmitz, 2012). We placed four different landmarks on each intersection. These were common German nouns with the first letter ranging from “A” to “L” (12 decision points). These nouns always consisted of six letters and two syllables. At each intersection every word contained the same initial letter. Four words on each of the 12 intersections resulted in a total of 48 different words as landmarks. For balancing purposes, each landmark word had to occur at every position at an intersection (*A,B,C*, and *D*) and had to be associated with each possible turning direction (left and right). We therefore created eight different routes for the initial path and, based on that, eight routes for the return path in order to control for sequential effects. An exemplary intersection in the allocentric condition is visualized in **Figure 8**.

##### Procedure

Before the first instruction for the main task was presented, subjects had to answer some demographic and exploratory questions. Thereafter, subjects were instructed to memorize the path, which was presented in a series of screenshots similar to **Figure 8**. For each intersection, they were asked to *memorize at least one landmark and the associated turn direction*. Depending on the condition subjects were pseudo-randomly assigned to one of the following additional instructions:

• *Instruction 2a:* the task was not only to remember the path but also to subsequently provide a *route description of the learned path* for another person also unfamiliar with this environment.
• *Instruction 2b:* the task was not only to remember the path but also to subsequently provide a *route description of the return path* (reverse learning order) for another person also unfamiliar with this environment.

After Instruction 2, the learning phase started, in which the route of 12 screenshots (i.e., 12 intersections) had to be learned. Finally, when the learning phase was over, subjects were asked to provide a route description of the learned path (*Instruction 2a*) or of the appropriate return path (*Instruction 2b*) for the test phase. Subjects entered the landmarks and directions into free text fields.

#### Results

**FIGURE 9:**
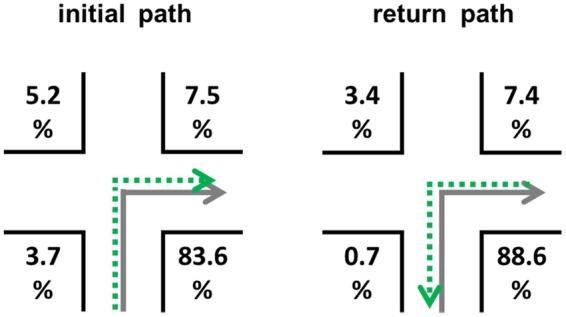
**Distribution of correct landmark-direction combinations for the initial path (left) and return path (right) in the allocentric perspective**. The gray solid arrows indicate the learning condition, while the green dotted arrows indicate the direction at retrieval. Please note that numbers do not necessarily add up to 100 due to rounding.

With a total of 62 subjects and 12 decision points per subject, 744 possible landmarks could have been named correctly. Our descriptive results showed that subjects described 411 landmarks correctly. The different landmark words were used equally often [χ*^2^*(47) = 31.511, *p* = 0.960]. A total of 283 correct combinations of landmarks and directions were reported by subjects. On average, for the initial path 42.95% (*SEM* = 6.81) of all possible landmark-direction combinations were correctly reported. For the return path, this occurred only in 34.49% (*SEM* = 6.28) of the cases. This difference is statistically insignificant [*t(*60*)* = 0.886, *p* = 0.379]. **Figure 9** shows the chosen positions of the correctly described landmarks in combination with the correct directional information.

Taken together a significant landmark position preference is visible [χ*^2^*(3) = 197.675, *p* < 0.001; deviation from an equal distribution]. These position preferences differ significantly [χ*^2^*(3) = 15.277, *p* < 0.001] from each other. In both cases the position *before the intersection in the direction of turn* (relative position depending on the direction of travel) is by far the most preferred one (initial path: 83.6%, return path: 88.6%).

#### Discussion Experiment 3a

The difference between performance for the initial path and return path is about 25%. This difference was, however, not significant due to a large variance in the data. Therefore, we cannot provide support for the first hypothesis: describing route directions for the initial path seems not to lead to better recollection than describing the return path. This is not what we intuitively expected and what has previously been demonstrated empirically for wayfinding performance (Gillner and Mallot, 1998; Wiener et al., 2012; Hinterecker et al., 2014).

For the second hypothesis some empirical evidence has been found. In both conditions, initial and return path, landmarks located at the position before the intersection and in the direction of turn were used for route descriptions in about 85% of the cases. This strengthens previous findings on the initial path (Röser et al., 2012a,b) and further supports the structural importance of this position during a landmark-based wayfinding process (Röser, 2015), also for the return path.

So far we concentrated on the allocentric perspective and Experiment 3b will now be realized in the egocentric perspective. Then, we will also be able to provide (comparison) data for the third hypothesis.

### Experiment 3b – Landmark Location Preferences from an *Egocentric* Perspective

#### Methods

##### Subjects

A total of 191 individuals (142 females, 42 males, seven did not provide gender information) participated. The mean age was 24.53 years (range = 17–77). They were recruited via a circular e-mail at the Justus Liebig University Giessen. Sixty-four percent (123 subjects) indicated to have a high-school diploma or similar. For the analysis a total of 88 could be included, since the others dropped out during the experiment and did not complete it. The remaining sample consisted of 76 females and 12 males with a mean age of 23.76 (range = 17–42). The percentage of high-school diploma or similar increased to 73%. All subjects provided informed consent and participation was voluntary without any compensation.

##### Materials

**FIGURE 10:**
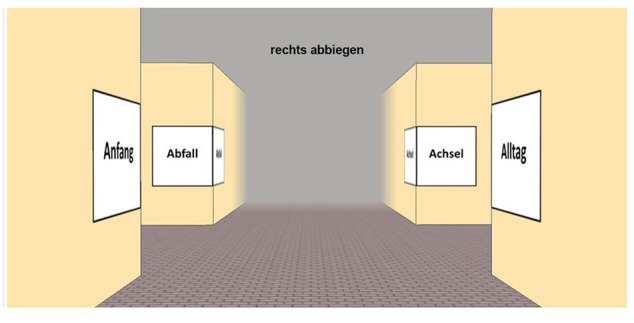
**Exemplary intersection in the egocentric perspective; arrangement and words identical to **Figure 8**. “Rechts abbiegen” indicates the directional information (turn right)**.

The materials (content) of Experiment 3b were identical to those of Experiment 3a, but now presented in an egocentric perspective (**Figure 10**).

##### Procedure

The procedure of Experiment 3b was identical to that of Experiment 3a, except for the perspective change. Now, subjects learned the route and landmark information in an egocentric perspective. The position of the subjects was the same at each intersection: in the middle of the path with a fixed distance to the center of the intersection. The eye-height was again set to 170 cm and the viewing direction was straight ahead.

#### Results

**FIGURE 11:**
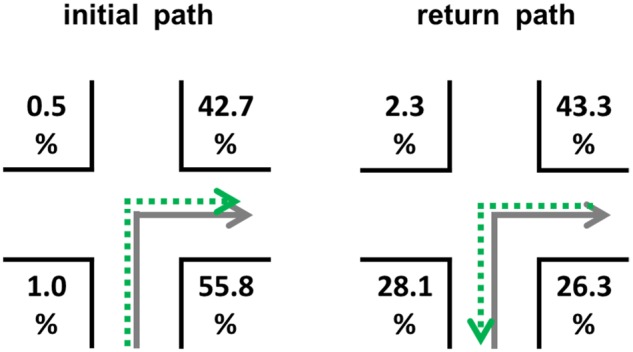
**Distribution of correct landmark-direction combinations for the initial path (left) and return path (right) in the egocentric perspective**. The gray solid arrows indicate the learning condition, while the green dotted arrows indicate the direction at retrieval. Please note that numbers do not necessarily add up to 100 due to rounding.

With a total of 88 subjects and 12 decision points per subjects, 1056 possible landmarks could have been named correctly. Our descriptive results showed that subjects described 514 landmarks correctly. The different landmark words were used equally often [χ*^2^*(47) = 39.732, *p* = 0.765]. A total of 370 correct combinations of landmarks and direction were provided. On average, for the initial path 40.06% (*SEM* = 5.84) of all possible landmark-direction combinations were correctly reported. For the return path this occurred in only 28.94% (*SEM* = 5.14) of the cases. This difference is statistically insignificant [*t*(86) = 1.292, *p* = 0.200]. **Figure 11** shows the chosen positions of the correctly described landmarks in combination with the correct directional information. Taken together a significant landmark position preference is visible [χ*^2^*(3) = 57.769, *p* < 0.001; deviation from an equal distribution]; these position preferences differ significantly [χ*^2^*(3) = 60.532, *p* < 0.001] from each other. In general, landmarks initially located in the direction of the turn were described more often. However, for the return path, landmarks located at *C*, the position *before the intersection opposite to the direction of turn* (from the perspective of the return path *behind the intersection and in the direction of turn*), are used for correct route descriptions in 28.1% of the cases (**Figure 11**).

#### Discussion Experiment 3b

As it was the case in Experiment 3a, we did not obtain evidence for the first hypothesis: the number of correct landmark-direction combinations was not higher for the initial path compared to the return path. The expected group differences were only visible on a descriptive level.

For the second hypothesis only partially empirical evidence has been obtained. In the initial path condition the positions in the direction of turn were the preferred ones. For the return path only the position *behind the intersection and opposite to the direction of turn* was hardly ever chosen. Position *B* was preferred and so were *C* and *D*. The findings for the initial path (highest preferences for landmarks at positions in the direction of turn) underline previous findings (Röser et al., 2012a,b). The slight shift within the position preferences for the return path is a new finding (theoretically addressed in the section “Theoretical Assumptions on the Return Path”). Interestingly, subjects now also describe landmarks located at the position *before the intersection and opposite to the direction of turn*, which from the perspective of the return path are located behind the intersection and in the direction of turn.

### Discussion Experiment 3

Previous experiments supported the hypothesis of higher performance for an initial path in comparison to a return path (e.g., Experiment 1). The current experiment used free recall and revealed only a descriptive tendency into the assumed direction. The reason for why these large differences were insignificant may be attributed to the occurrence of a large variance in this rather difficult task of free landmark-based route description in comparison to simple cued retrieval tasks.

Does the position preference depend on the wayfinder’s task? We hypothesized that this is not the case. In Experiment 3a, we found that position *D* was preferred in both the initial and the return path. It was chosen at least five times more often than the other three positions taken together. In Experiment 3b, *B* and *D* were preferred when subjects were instructed to describe the initial path beforehand. Interestingly, in the return path condition, subjects preferred next to *B* and *D* also *C*. While the importance of *B* and *D* has been discussed extensively, we need to take a closer look to *C*. How can the increasing usage of position *C* be explained? This position *before the intersection and opposite to the direction of turn* marks the position *behind the intersection and in direction of turn* from the perspective of the return path. This means that the preference shift from position *D* to position *C* is attributed to a mental transformation of perspective. In the Section “Theoretical Assumptions on the Return Path,” we predicted that positions *B* and *D* should be the ideal ones when describing a return path, since the given structure during encoding and especially visual attention should determine encoding efficacy. However, the empirical results partially contradict this theoretical assumption. It may not account for the observed shift from position *D* (which should still be the most preferred one) to position *C*.

The difference of the landmark location preference between the allocentric and egocentric perspective could be described in terms of viewpoint-based salience (Röser, 2015). In an egocentric perspective the landmarks differ within the degree of distance and how much of a landmark is visible (visible part). This influences subjects’ preferences and leads to an increase of the landmark position behind the intersection and in the direction of turn (hypothesis 3). Our data fit very well with the assumption and findings of our landmark salience model (Röser et al., 2012a; Röser, 2015). This model includes the structural salience, moderated by the viewpoint-based salience and would predict for the current experiment that both positions in the direction of turn are used for creating a route description most and equally often. However, the differences of landmark usage in route descriptions between an initial and a return path in the egocentric perspective is not considered in this model to date.

To consider the task of finding the return path a new factor should be implemented in the model. We label this factor *task*, which is in accordance with one factor of Caduff and Timpf’s (2008) landmark salience model. Their model differentiates between different *traveling tasks*, such as sightseeing or commuting. We extend this factor with the task *direction of travel*. This includes mental rotation/transformation of view directions and traveling direction, as well as lingual requirements.

## General Discussion

In this study, we focused on structural landmark salience in a certain context. This context consisted of theoretical issues that are of interest when investigating cognitive processes in human landmark-based wayfinding: finding the return path, landmark positions and the problem of perspective. We therefore elaborated theoretical assumptions in regards to these problems. The key question has always been: “Which of four landmark positions at an intersection are preferable when it comes to finding the return path?” We emphasized the role of perspective, that is how the encoding and retrieval perspective (egocentric versus allocentric) influence route learning. Other factors involved were direction specificity of verbal information (e.g., “turn right in front of *A”* versus *“*turn in direction of *D”*) and invariance of positions (e.g., *D* remains the *direction of turn before the intersection* in both the initial and return path).

We began our work by showing that recalling a return path is not the same as recalling an initially learned path in the same direction. According to this finding, a mere generalization of results from research testing an initial path on finding the return path seems to be inappropriate. The cognitive processes involved in the initial path and the return path seem to be somehow different or additional processes are required for the return path (which is more likely from our point of view). Other cognitive mechanisms and neural structures need to be considered. The findings of Experiment 1 underline this presumption and give reason to further explore the topic with focus on landmark positions.

In Experiment 2, we addressed the topic of structural landmark salience (in regards to invariance of positions). Further, we addressed the question of how different learning modalities (encoding perspectives), such as verbal description and map learning, influence our wayfinding performance. We found position effects but no effects for learning modality. Our mixed findings for the first two experiments show how important it is to consider a multifactorial approach. Other studies indicate that a distinction between low and high performers would have made sense (Baumann et al., 2011). Inter-individual spatial abilities, working memory capacities and different preferred strategies have to be taken into account. This notion is supported by recent findings of Pazzaglia and Moè (2013) showing different performances in map learning for people with different cognitive styles (visualizer versus verbalizer; Richardson, 1977) and spatial abilities (mental imagery and mental rotation). Thus, the next step in our research has to be not only to focus on the landmark positions, mental and verbal transformations for the return path, but also on cognitive styles (Pazzaglia and Moè, 2013) and personal (preferred) strategies (e.g., Kato and Takeuchi, 2003).

In another study, the results revealed that subjects are indeed performing better with unspecific route directions compared to specific spatial wording (Hinterecker et al., 2014). However, if they were later asked to generate a verbal description of the path, they preferably made use of spatial words (left/right). Therefore, the distinction between specific and unspecific information is appealing and the unspecific information at present seems to be the better one. But, humans (at least in western cultures) preferably use spatial terms, since they were learned and used throughout their lifetime. This issue needs to be investigated further in order to make a clear distinction between specific and unspecific spatial information with respect to landmark position and independence of cultural origin.

In Experiment 3, we tried to show that landmark position preferences differ between initial and return path by using a free recall paradigm. While descriptive results showed a tendency in the hypothesized direction, the inferential statistic results remained insignificant due to a large variance in the data. Thus, using this paradigm, we were not able to show such an effect. Interestingly, we were able to show that the position preference does not depend on the wayfinder’s task. In other words, the encoding direction is decisive when it comes to optimal landmark positions. Because subjects encoded the initial path, the perspective and the spatial configuration of landmarks during this phase were the most important. We therefore need to reject the idea that the invariance of a landmark position plays an important role. Otherwise the results would have shown that *A* is much preferable.

The fact that we used different measures helped us to gain insight into the cognitive processes involved in finding the return path. Findings that were based on route continuation measures and measures of landmark recall have to be interpreted with the following in mind. Route continuation measures (cued wayfinding) and landmark recall (free recall) do not rely on identical mental processes. For instance, route continuation does not solely rely on recognition because pure recognition would result in chance level performance in wayfinding. Such a measure relies on cued recall (pairs of landmarks and directions) and/or serial recall (list of route directions). This seems to be logical since not every environment contains salient objects the way urban environments do. This underlines the very fact that wayfinding also works without landmarks. The extent to which each of these two strategies is used is observer-dependent and can account for performance differences in landmark recognition even when the strategies are self-reported (Karimpur and Hamburger, 2016). Another limitation is that several factors influence the strategy choices based on route descriptions. For instance, Brunyé et al. (2015) examined the influence of conflicting information (e.g., wrong direction/reference point) and different sources (e.g., human, GPS systems, etc.). Their findings suggest that subjects prefer to use landmark-based strategies with information provided by humans and direction-based strategies with GPS information indicating that the respective opposite could be flawed. Therefore, further studies should also take these findings into account and consider the source of information as a limiting factor. Finally, we should bear in mind that there is also an alternative interpretation of our data. In our effort to use different methods, the differences we obtained could also be due to differences in task demands. For example, in Experiment 3, we instructed subjects beforehand to provide a verbal description. In fact, this could also explain how we found a shift toward C when it came to landmark position preferences in the return path for the egocentric perspective.

**FIGURE 12:**
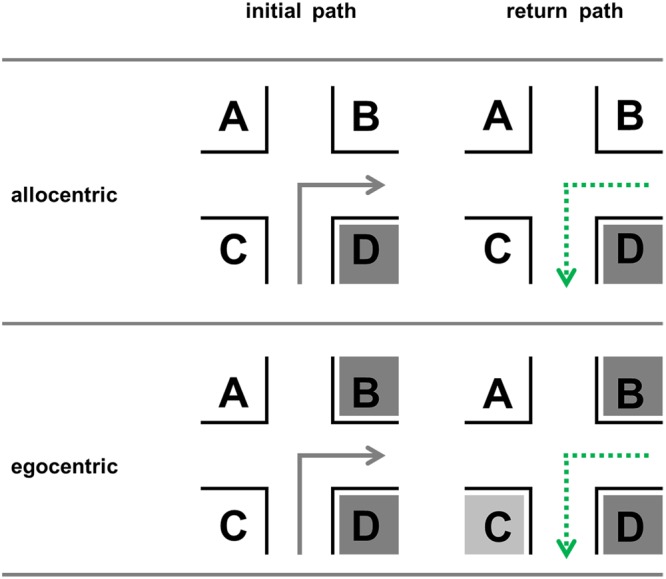
**Change of position preferences.** Position *D* represents the ideal position in all conditions. In the egocentric conditions, the positions in the direction of turn are used most often to describe an initial path correctly (*B* and *D*), while in the return path condition also position *C* is used in a substantial number of correct reports (compare with theoretical predictions; **Figure 2**).

Now what is the most preferred landmark position? In sum it can be said that *D* is the best position in general. In an allocentric perspective it is by far the most prominent landmark location. Especially when encoded from an egocentric perspective, we could also say that the positions in direction of the turn, namely *B* and *D*, are the best positions. However, our data also revealed something interesting: in everyday life perspective (egocentric) there is a shift toward *C* (**Figure 12**), which is a variant position. This means that it mentally as well as verbally needs to be transformed, i.e., the position before the intersection and opposite to the direction of turn has to be represented as behind the intersection and in the direction of turn for the return path and so on (**Figure 12**). Why this increased cognitive load is voluntarily chosen by people remains an open question for future research.

## Conclusion

As can be seen from our theoretical assumptions and empirical findings, more sophisticated research is required within this context. We offered a few important issues, e.g., structural importance, visibility, language, and mental transformation, which need to be investigated more thoroughly in landmark-based wayfinding. So far we did not focus on brain imaging and neural correlates of landmark-based wayfinding (e.g., Janzen and van Turennout, 2004). But, investigating the cognitive processes of how we learn and encode initial pathways and how we later transform them into new routes (especially return paths) is also of relevance for the neuroscientific branch of this research. Thus, our findings and assumptions about the return path make up for valuable interdisciplinary future cognitive research.

## Ethics Statement

The experiments were conducted in accordance with the Declaration of Helsinki and were approved by the local ethics board of the Justus Liebig University Giessen (2014-0017). Participants had to provide informed written consent. They were informed that participation was voluntary and that they could terminate the experiment at any time without any reason and any negative consequences.

## Author Contributions

HK and FR have been involved in designing the study, data collection, data analysis, and writing the manuscript. KH has been involved in designing the study, data analysis, and writing the manuscript.

## Conflict of Interest Statement

The authors declare that the research was conducted in the absence of any commercial or financial relationships that could be construed as a potential conflict of interest.
